# Factors Affecting the Intraluminal Therapy for *Helicobacter pylori* Infection

**DOI:** 10.3390/microorganisms10020415

**Published:** 2022-02-11

**Authors:** Cheng-Yu Ho, Ting-Wen Liu, Yang-Sheng Lin, Yen-Po Chen, Ming-Jen Chen, Horng-Yuan Wang, Tai-Cherng Liou

**Affiliations:** 1 Division of Gastroenterology, Department of Internal Medicine, MacKay Memorial Hospital, Tamsui, New Taipei City 25173, Taiwan; chanyo123@gmail.com (C.-Y.H.); drchen87@gmail.com (Y.-P.C.); 2 Department of Medicine, MacKay Medical College, New Taipei City 25245, Taiwan; carlos60531@gmail.com (T.-W.L.); u802005@gmail.com (Y.-S.L.); mingjen.ch@msa.hinet.net (M.-J.C.); mmh4013@gmail.com (H.-Y.W.); 3 MacKay Junior College of Medicine, Nursing, and Management, Taipei 10449, Taiwan; 4 Department of Internal Medicine, MacKay Memorial Hospital, Taipei 10449, Taiwan; 5 Division of Gastroenterology, Department of Internal Medicine, MacKay Memorial Hospital, Taipei 10449, Taiwan

**Keywords:** *Helicobacter pylori*, gastric juice, acidity, endoscopy, therapy, risk factors

## Abstract

*Helicobacter pylori* (*H. pylori*) can be eradicated immediately while conducting an endoscopic examination. The eradication rate of intraluminal therapy for *H. pylori* infection (ILTHPI) is 53.7% (51/95) via local application of single-dose medicament containing amoxicillin, metronidazole, and clarithromycin. We aimed to evaluate factors affecting ILTHPI and to assess the efficacy among single antibiotics, and compared our results with combined antibiotics. We enrolled *H. pylori*-infected treatment-naïve symptomatic patients; 95 completed triple-antibiotic ILTHPI were evaluated for risk factors, along with 60 completed mono-antibiotic ILTHPI containing amoxicillin, clarithromycin, or metronidazole in each of the 20 patients. Univariate analysis revealed the significant influence of BMI (OR: 1.15; 95% CI: 1.03–1.27, *p* = 0.011) and gastric juice pH (OR: 1.35; 95% CI: 1.16–1.58, *p* = 0.0001). Logistic regression analysis also showed significant influence of gastric juice pH (OR: 1.30; 95% CI: 1.10–1.54, *p* = 0.002). The eradication rate of mono-antibiotic ILTHPI is significantly lower than triple-antibiotic ILTHPI (11.7% vs. 53.7%; *p* < 0.0001; α = 0.05, power = 1.0). The efficacy was 20% (4/20) for metronidazole, 10% (2/20) for amoxicillin, and 5% (1/20) for clarithromycin. In conclusion, the level of gastric juice pH is a crucial factor affecting the ILTHPI. The detection of gastric juice pH and selection of optimal intraluminal medicaments are important. Further studies with combined antibiotics for ILTHPI, perhaps metronidazole-containing medicaments, are recommended.

## 1. Introduction

About half of the global population are infected with Helicobacter pylori (*H. pylori*) [1], which leads to gastric inflammation, peptic ulcer disease, gastric cancer, and mucosal associated lymphoid tissue lymphoma of the stomach [2]. Up to 89% of all gastric cancer and about 15% of global cancer burden are ascribed to the infection of *H. pylori* [3]. Peptic ulcer disease can be relieved and the risk of gastric cancer decreased after the eradication of *H. pylori* [2,4]. However, due to the global emergence of *H. pylori* antibiotic resistance [5], some oral antibiotic regimens are no longer appropriate as the first-line treatment due to the <80% eradication rate in most areas. Several strategies based on a new combination of antibiotics have been developed [2,6,7]. However, the rates of primary and dual resistance keep going up due to the prolonged usage of multiple-dose antibiotics [8,9] which calls for alternative novel therapy [10]. Owing to the special gastric milieu, no single-dose oral agent can eradicate *H. pylori* immediately. However, *H. pylori* can be eradicated immediately via single-dose medicaments applied while conducting an endoscopic examination [11]. The process of intraluminal therapy for *H. pylori* infection (ILTHPI) contain “the control of stomach pH, irrigating the gastric mucus, and the application of a single-dose antimicrobial medicaments”. After ILTHPI with medicament containing combined antibiotics (amoxicillin, clarithromycin, and metronidazole), the immediate eradication rate is 53.7% (51/95) [11], and significantly higher (72%; 36/50) for patients with gastric juice pH at or above 4 [12]. However, besides the intragastric pH, the factors influencing effectiveness of ILTHPI are unclear. In addition, whether a single antibiotic can achieve successful ILTHPI, the contribution of each antibiotic, and the potential secondary antibiotic resistance associated with ILTHPI all remain unknown. The primary aim of the current study was to evaluate factors affecting the ILTHPI. The secondary aim was to assess the efficacy and the potential secondary antibiotic resistance related to the mono-antibiotic ILTHPI for three antibiotics (including amoxicillin, clarithromycin, and metronidazole).

## 2. Materials and Methods

### 2.1. Patients

#### 2.1.1. Intraluminal Triple Antibiotic Therapy

From April 2017 to December 2017, 100 patients completed the ILTHPI in a clinical trial (NCT03124420) with medicament containing powders of amoxicillin (3 g), clarithromycin (1 g), and metronidazole (2 g) as detailed in our previous report [11]. The clinical characteristics of enrolled patients and endoscopic findings were recorded. Five patients were lost to follow up, fifty-one per-protocol patients achieved successful ILTHPI, and forty-four patients failed ILTHPI and completed one-week (*n* = 21) or two-week (*n* = 23) standard triple therapy. The possible clinical factors influencing the effectiveness of triple antibiotic ILTHPI were evaluated in the current study. The factors evaluated for their effect on predicting successful ILTHPI included age, gender, body mass index (BMI), serum anti-*H. pylori* immunoglobulin G (HPIgG) (LIAISON *H. pylori* IgG, DiaSorin Inc., Stillwater, MN, USA), and the delta over baseline (DOB) value for the ^13^C-UBT using 75 mg ^13^C urea (Helico-BT K.M., HWANG’S Pharmaceutical Co. Ltd., Taiwan). We analyzed, with a nondispersive infrared spectrometer, the type of disease (Type 2 diabetes mellitus, dyslipidemia, renal function, Anemia), personal habits (smokers, users of alcohol, tea, coffee, NSAID, steroid, statin), and the endoscopic findings (gastric juice pH, gastric ulcer and/or duodenal ulcer, inflammation of cardia, positive ultrafast 300 quick test (UFT300; BIOHIT Oyj, Helsinki, Finland). The renal function was estimated based on MDRD calculation for value of estimated glomerular filtration rate (eGFR). eGFR (mL/min/1.73 m^2^) = 175 × creatinine (mg/dL)^−1.154^ × age^−0.203^ × 0.742 (if female). An eGFR more than 90 mL/min/1.73 m^2^ was classified as chronic kidney disease (CKD) stage 1. An eGER of 60–89, 30–59, or 15–29 mL/min/1.73 m^2^ was classified as CKD stage 2, 3, or 4, respectively. An eGFR below 15 mL/min/1.73 m^2^ was classified as CKD stage 5.

#### 2.1.2. Intraluminal Mono-Antibiotic Therapy

**Figure 1:**
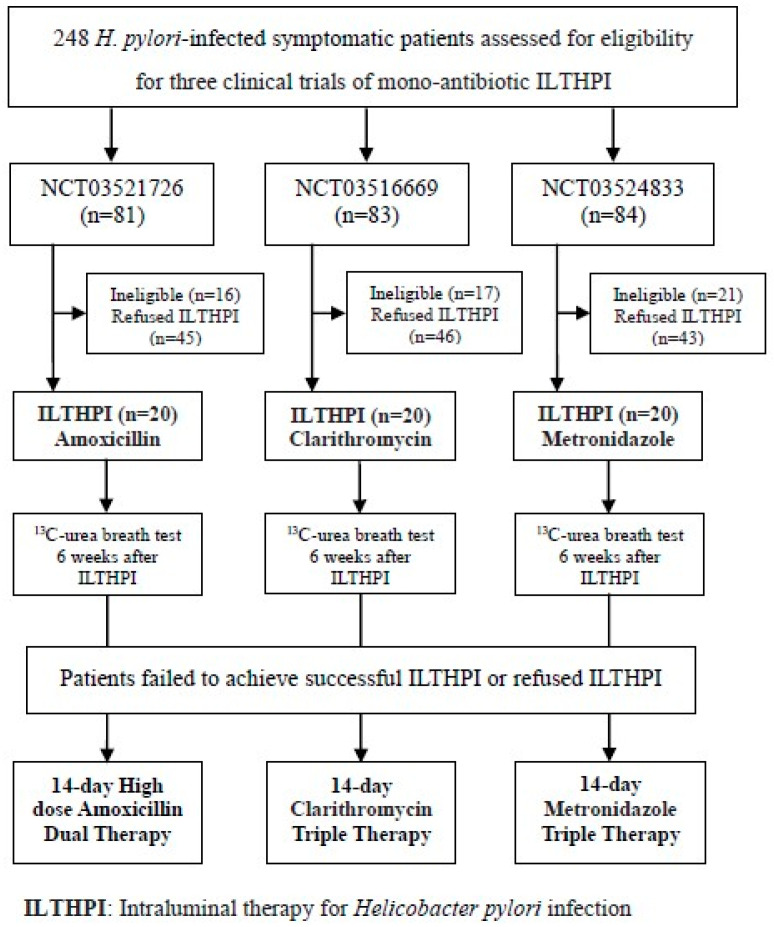
Study flow chart.

Three consecutive prospective pilot studies were conducted between May 2018 and December 2019 to assess the efficacy of mono-antibiotic ILTHPI for three antibiotics including amoxicillin, clarithromycin, and metronidazole. A total of 248 ^13^C-UBT positive *H. pylori*-infected patients, ages between 20 and 75, were evaluated for eligibility of ILTHPI with the same inclusion criteria as our previous study [11]. Fifty-four ineligible patients were excluded, including: (1) seven contraindication of endoscopy; (2) two previous gastric surgery; (3) two deformity, stenosis, or obstruction of gastroduodenum; (4) two gastroduodenal malignancy; (5) six reinfection of *H. pylori*; (6) three use of antibiotics or bismuth in the previous four weeks; (7) twenty-one use of PPI or H2-blockers in the previous two weeks; (8) two previous allergic history to medicaments used; (9) two pregnant or lactating women; (10) six severe concurrent diseases (Two CKD stage 4, One CKD stage 5, One decompensated liver cirrhosis, Two malignancy); and (11) one inability or refused to give informed consent. As shown in Figure 1, a total of 194 *H. pylori*-infected treatment-naïve patients were enrolled for endoscopic examination, detection of gastric juice pH, and concomitant mono-antibiotic ILTHPI before oral antibiotic therapies. Among them, 134 patients agreed with oral antibiotic therapies but refused ILTHPI, 60 patients received mono-antibiotic ILTHPI in groups of 20 patients, respectively, for 3 clinical trials

(NCT03521726/NCT03516669/NCT03524833). Patients failed to achieve successful ILTHPI or refused ILTHPI in each clinical trial and received oral antibiotic therapy containing the same antibiotic used in the ILTHPI. The efficacy and adverse event of each mono-antibiotic ILTHPI were evaluated. The eradication rates and adverse events of oral antibiotic therapies were also compared between patients failed to achieve successful ILTHPI and patients without ILTHPI in each clinical trial. From an ethical standpoint, given that the eradication rates of mono-antibiotic ILTHPI were still unknown, we allowed the initially enrolled patients, who agreed with the ILTHPI and signed informed consents in out-patient clinic, to decline the ILTHPI before the endoscopic examinations. In addition, the investigators still provide endoscopic examinations without ILTHPI, the detections of gastric juice pH, and the oral antibiotic therapies for those patients as scheduled. We enrolled a limited number of 20 patients in each clinical trial for mono-antibiotic ILTHPI and 134 patients, who declined ILTHPI, 45 patients received high dose amoxicillin dual therapy, and the remaining received amoxicillin–containing triple therapy (46 clarithromycin and 43 metronidazole). All participants provided written informed consent, and the three pilot studies were approved by the Institutional Review Board of our hospital (IRB numbers: 17MMHIS096, 17MMHIS097, 17MMHIS098 on 24 January 2018).

ILTHPI, intraluminal therapy for Helicobacter pylori infection.

### 2.2. Methods

#### 2.2.1. Detection of Gastric Juice pH

Clinical characteristics and endoscopic findings for 194 enrolled patients were recorded. Levels of gastric juice pH were also detected for all patients. All of the procedures of mono-antibiotic ILTHPI in three clinical trials were performed by a single, experienced senior gastroenterologist, and were strictly followed as mentioned in our previous report [11]. “For all participants, a high-resolution electronic endoscope from Olympus Co. (Tokyo, Japan, GIF 260 or 290 series) was used for detection and interpretation of mucosal changes, such as ulceration or hyperemia” [11]. Levels of gastric juice pH were detected, and the methodology was detailed in our previous report [12]. “The pH of gastric juice was measured using two pH strips (Macherey-Nagel pH-Fix 0.0–6.0 and pH-Fix 4.5–10.0); each scaled in pH 0.5 intervals. Levels of gastric juice pH in each patient are classified into three ranges, including normal acidity (pH < 4.0), low level hypoacidity (pH 4.0 to 5.5), and high-level hypoacidity (pH ≥ 6.0)” [12].

#### 2.2.2. Intraluminal Therapy for *Helicobacter pylori* Infection

The procedure of ILTHPI was as follows: “Patients were administered two tablets of orally disintegrating lansoprazole (30 mg per tablet) before ILTHPI and another two tablets 8 to 10 h after ILTHPI. During ILTHPI, an endoscopic apparatus and washing pipe from Olympus Co. (Tokyo, Japan, EndoTherapy product name: PW-IL-1) were used to irrigate the gastric mucus with acetylcysteine (12 m/mL) solution to remove the mucus on the gastric mucosa. The total dosage of acetylcysteine was less than 140 mg/Kg” [11]. The medicament of mono-antibiotic ILTHPI for each study respectively contains 3 g of amoxicillin powder for suspension, 1 g of crushed enteric-coated clarithromycin tablets in powder forms, or 2 g of crushed metronidazole tablets. “Each single antibiotic powder was mixed with 60 mL (6 g) sucralfate suspension and 120 mL of distilled water and was applied to all surfaces of gastric mucosa and the mucosa of duodenal bulb as evenly as possible using the same washing pipe. During the endoscopic examination and ILTHPI, each patient was sedated with intravenous midazolam (5 mg). Vital signs were closely monitored using a physiological monitor, and the safety profiles were strictly followed as defined in our previous report. After ILTHPI, patients were asked to rest for 30 min for the effects of sedation to wear off before leaving. However, they were allowed to take meals if they did not experience abdominal discomfort” [11].

#### 2.2.3. Oral Antibiotic Therapy for Patients Failed ILTHPI

The post-ILTHPI follow-ups were similar as our previous studies.” The post-ILTHPI follow-ups of the patients were conducted by other physicians. Patients were scheduled for consultation three to seven days after ILTHPI and all patients were asked to report presence or absence of adverse events during the consultation. The ^13^C-UBT was used to assess the successful eradication of *H. pylori* six weeks after ILTHPI. The eradication rate of ILTHPI was calculated for per-protocol patients, and those who failed to return for follow-up ^13^C-UBT were excluded. All patients with successful ILTHPI underwent subsequent stool *H. pylori* antigen tests (Artron *H. pylori* stool Ag rapid test; Artron Laboratory Inc., Burnaby, BC, Canada) four to six months after ILTHPI to exclude short-term recurrence of *H. pylori* infection” [11].

Patients who failed to achieve successful ILTHPI and those agreed with oral antibiotic therapies but declined ILTHPI received 14-day high dose amoxicillin dual therapy (rabeprazole 20 mg q.i.d. and amoxicillin 750 mg q.i.d.; NCT03521726), 14-day clarithromycin triple therapy (lansoprazole 30 mg b.i.d., amoxicillin 1000 mg b.i.d., and clarithromycin 500 mg b.i.d.; NCT03516669), or 14-day metronidazole triple therapy (lansoprazole 30 mg b.i.d., amoxicillin 1000 mg b.i.d., and metronidazole 500 mg b.i.d.; NCT03524833). Patient compliance and adverse events were assessed. *H. pylori* eradication were confirmed by ^13^C-UBT six weeks after the completion of treatment.

### 2.3. Statistical Analysis

We hypothesized that the average efficacy of each mono-antibiotic therapy should be lower than that of triple antibiotics therapy (53.7%; 51/95) as reported in our previous literature. Not to mention the synergistic effect, the expected average efficacy of these three mono-antibiotic therapies may be as high as one third of the efficacy of triple antibiotics therapy, i.e., 17.9% (53.7% × 1/3). We used the Sample Size Calculator (Kane SP. Sample Size Calculator. ClinCalc: available from https://clincalc.com/stats/samplesize.aspx, accessed 15 December 2021) to determine the minimum number of 20 subjects enrolled in each clinical trial in order to have sufficient statistical power to detect a difference (α = 0.05, power = 0.95). Both intention-to-treat (ITT) and per-protocol (PP) analyses were conducted. The ITT analysis included all patients who received therapies, and those who failed to do follow-up therapy or tests were considered treatment failures. The PP analysis excluded patients who failed to do follow-up therapy or tests as per protocol.

Values were expressed as mean ± standard deviation, unless otherwise indicated. Categorical data were compared using the χ^2^ test or Fisher’s exact test as appropriate. Continuous variables were expressed as mean ± standard deviation. Student’s *t*-test was used to compare the mean values of continuous variable. All reported *p*-values were based on two-sided tests and considered statistically significant if less than 0.05. Data were analyzed by using Stata 14 software (Stat Corp, College Station, TX, USA). All of the covariates were tested using unadjusted binary or ordinal logistic regressions, respectively. Corresponding 95% confidence interval (95% CI) were calculated for all estimates. A multiple logistic regression analysis was also performed to adjust potential confounders. Bonferroni correction was used to correct alpha inflation resulting from multiple regression model with a Bonferroni-corrected threshold of 0.05/7 = 0.007.

## 3. Results

### 3.1. Factors Affecting the ILTHPI

Table 1 showed the results of logistic regression analysis to factors affecting the ILTHPI. Univariate analysis revealed significant influence of the BMI (OR: 1.15; 95% CI: 1.03–1.27, *p* = 0.011) and gastric juice pH (OR: 1.35; 95% CI: 1.16–1.58, *p* = 0.0001). Multiple logistic regression analysis was also performed to adjust potential confounders including age, BMI, DOB value of ^13^C-UBT, Type 2 DM, smoking, gastric juice pH, and peptic ulcer disease (gastric ulcer and/or duodenal ulcer). The results also showed significant influence of the gastric juice pH (OR: 1.30; 95% CI: 1.10–1.54, *p* = 0.002) with a Bonferroni-corrected threshold of 0.05/7 = 0.007. However, there is no significant difference of the BMI (OR: 1.12; 95% CI: 0.998–1.27, *p* = 0.055). There are no significant influences (*p* > 0.05) of the other factors including age, gender, serum anti-HPIgG, DOB value for the ^13^C-UBT, type of disease (Type 2 diabetes mellitus, dyslipidemia, value of eGFR, Anemia), personal habits (smokers, users of alcohol, tea, coffee, NSAID, steroid, statin), and endoscopic findings (gastric ulcer and/or duodenal ulcer, inflammation of cardia, positive ultrafast 300quick test (UFT300) of cardia).

As shown in Figure 1 and Table 2, Table 3, Table 4 and Table 5, 60 patients completed the mono-antibiotic ILTHPI, and the remaining 134 patients received oral antibiotic therapies without ILTHPI (Group C). The average duration of ILTHPI was 8 min and 22 s, including the irrigation of gastric mucus (4 min and 36 s) and the application of medicaments (3 min and 25 s). The demographic and endoscopic characteristics, the distribution in the ranges of gastric juice pH level, and the overall efficacy and adverse events of mono-antibiotic ILTHPI in 60 patients (Group A) are compared with the results of triple antibiotic ILTHPI in 100 patients (Group B) reported in our previous study [11].

### 3.2. Characteristics of H. pylori Infected Patients

Table 2 showed the clinical characteristics of patients with mono-antibiotic ILTHPI (Group A), triple antibiotic ILTHPI (Group B), and those who received oral antibiotic therapies without ILTHPI (Group C). There are no significant differences (*p* > 0.05) in any of the demographic characteristics among patients in Group A, Group B, and Group C. There are also no significant differences (*p* = 0.624) in endoscopic findings among Group A, Group B, and Group C regarding the proportions of gastritis, peptic ulcer disease, or normal appearance. Subclassification of endoscopic gastritis also revealed no significant differences (*p* = 0.352) among the three groups regarding the distributions of endoscopic features of gastritis (hyperemic mucosal change) in antrum, corpus, and cardia. The levels of gastric juice pH were classified into three arranges for patients with *H. pylori* infection. There are no significant differences (*p* = 0.425) in the distributions of pH levels among Group A, Group B, and Group C.

### 3.3. The Efficacy and Adverse Event of ILTHPI

Table 3 revealed the efficacy and adverse event of intraluminal mono-antibiotic and triple antibiotic therapy for *H. pylori* infection. All of the 60 patients completed the mono-antibiotic ILTHPI with good safety profiles and without violation of any of the safety indicators as defined in our previous report [11]. Seven of the sixty patients (11.7%; 95% CI: 4.8–22.6%) achieved successful eradication with negative C^13^-UBT six weeks after ILTHPI, and the subsequent stool *H. pylori* antigen tests were all negative four to six months after ILTHPI, no shot-term recurrence was observed. The eradication rate of ILTHPI is significantly lower in patients with mono-antibiotic ILTHPI as compared with those of triple-antibiotic ILTHPI (53.7%; 95% CI: 43.7–63.4%) (*p* < 0.0001; α = 0.05, power = 1.0). The efficacy of each pilot study of mono-antibiotic LTHPI was 20% (4/20) for metronidazole, 10% (2/20) for amoxicillin, and 5% (1/20) for clarithromycin. Although not statistically significant, the eradication rate of metronidazole is higher than for amoxicillin and clarithromycin. Only 1 of 20 patients (5%) in the study of metronidazole subgroup suffered from nausea for 6 to 8 h on the day of ILTHPI. The severity of adverse events after ILTHPI was mild as defined by de Boer et al. [13]. No adverse event was observed in the other subgroups including amoxicillin and clarithromycin. There is no significant difference for the overall incidence rate of adverse event between mono-antibiotic ILTHPI (1.7% [1/60]) and triple antibiotic ILTHPI (6% [6/100]) (*p* = 0.257).

Table 4 showed the overall eradication rate of successful mono-antibiotic ILTHPI plus oral antibiotic therapy for patients failed ILTHPI containing the same antibiotic in three clinical trials. All of the 53 patients failed to achieve successful mono-antibiotic ILTHPI completed the rescue oral antibiotic therapies with good compliance (took ≥ 80% drugs) and all received subsequent ^13^C-UBT. The eradication rates, either for ITT or PP, were 88.9% (16/18; 95% CI: 66.0% to 98.1%) for 14-day high dose amoxicillin dual therapy, 94.7% (18/19; 95% CI: 73.5% to 99.9%) for 14-day clarithromycin triple therapy, and 75.0% (12/16; 95% CI: 50.0% to 90.3%) for 14-day metronidazole triple therapy.

Table 5 showed the efficacy of oral antibiotic therapy for patients failed ILTHPI and without ILTHPI in three clinical trials. For the 134 patients without ILTHPI, all of them completed oral antibiotic therapies with good compliance (took ≥ 80% drugs); four patients dropped out without subsequent ^13^C-UBT (one in amoxicillin subgroup, two in clarithromycin subgroup, and one in metronidazole subgroup). The ITT and PP eradication rates were 84.4% (38/45; 95% CI: 70.9% to 92.6%) and 86.4% (38/44; 95% CI: 72.7% to 94.8%) for 14-day high dose amoxicillin dual therapy, 80.4% (37/46; 95% CI: 66.6% to 89.6%) and 84.1% (37/44; 95% CI: 70.3% to 92.4%) for 14-day clarithromycin triple therapy, 72.1% (31/43; 95% CI:57.2% to 83.4%) and 73.8% (31/42; 95% CI: 58.8% to 84.8%) for 14-day metronidazole triple therapy. The ITT and PP eradication rates of each oral antibiotic therapy were higher for patients with failed ILTHPI than those without ILTHPI, although there are no significant differences between them (*p* > 0.05). In addition, after clarithromycin ILTHPI, the ITT and PP eradication rates of 14-dayclarithromycin triple therapy for those failed ILTHPI are still higher than 90%.

The severities of adverse events of all oral antibiotic therapies were mild in classification as defined by de Boer et al. [13]. The overall incidence rates of adverse events of each oral antibiotic therapy are also similar comparing patients with failed ILTHPI and those without ILTHPI regarding 14-day high dose amoxicillin dual therapy (16.7% [3/18] vs. 20% [9/45]; *p* = 1.000), 14-day clarithromycin triple therapy (31.6% [6/19] vs. 32.6% [15/46]; *p* = 1.000), and 14-day metronidazole triple therapy (25.0% [4/16] vs. 27.9% [12/43]; *p* = 1.000).

## 4. Discussion

The indications for *H. pylori* eradication and endoscopic examination are diverse due to the geographic differences in the availability of medications and endoscopy, the prevalence of gastric cancer, and the health insurance systems involved. *H. pylori* is one of the significant contributors of gastritis leading to gastric cancer, especially combined with chronic alcohol consumption and the usage of capsaicin [14,15]. In Taiwan, our national health insurance (NHI) system provides entire coverage for medications used to eradicate *H. pylori* and endoscopy for pretreatment screening and posttreatment surveillance due to the high prevalence of *H. pylori* infection and gastric cancer [16,17]. With global economic growth, a gradual aging population, and the establishment of the NHI, guidelines for the indications of endoscopy and therapeutic strategies for *H. pylori* eradication are changing and steadily including the early detection of gastric cancer [2,6,7].

Since ILTHPI achieves concomitant eradication of *H. pylori* during endoscopic examination and eliminates subsequent multiple-dose oral antibiotic therapies, which reduce the burdens of treatment for individuals and global NHI systems. The ILTHPI could provide a 72% (36/50) chance of eradication for patients with gastric juice at or above 4 [12]. To achieve an appropriate (>80%) or optimal (>90%) eradication rate of ILTHPI, factors affecting the intraluminal therapy for *H. pylori* infection should thus be determined. Our study has limitations. First, we enrolled *H. pylori*-infected treatment-naïve patients and excluded some patients who may be suitable for ILTHPI, including the six *H. pylori* reinfected patients and the 21 users of PPI or H2-blockers. Second, we enrolled patients for ILTHPI based on patient’s willingness, rather than randomization. However, there are no significant difference among patients in Group A, Group B, and Group C regarding the demographic characteristics, the ranges of gastric juice pH, and the endoscopic findings (Table 2). Third, we only enrolled a limited number of 20 patients in each clinical trial for mono-antibiotic ILTHPI. Due to the small sample size in our studies, the differences in the geographical prevalence, the antibiotic resistance, and the therapeutic policies of *H. pylori* infection, larger sample-size studies in different geographical area are necessary to authenticate our findings. Furthermore, antibiotic resistance is an important factor affecting the eradication rate, however, the antibiotic susceptibility tests (AST) were not conducted in our medical center and in most medical institutions due to the possibility of false negatives and problems of clinical applicability [18].

Antibiotic resistance is regarded to be the most crucial factor affecting the eradication of *H. pylori*. However, a recent meta-analysis displayed that “Susceptibility-guided treatment is not better than empirical treatment of *H. pylori* infection in first-line therapy if the most updated quadruple regiments are empirically prescribed” [18]. Since our study were conducted in 2017 when the primary resistance of *H. pylori* to clarithromycin is less than 15% in Taiwan [19], the AST may be exempted for the triple antibiotic ILTHPI in our study. However, due to the global increasing primary resistance rate for clarithromycin, metronidazole, and levofloxacin [8,9], to achieve a higher eradication rate of ILTHPI, selection of optimal antibiotic in accordance with the geographical surveillance of antibiotic resistance or based on pretreatment molecular stool AST [20] is of extreme importance.

Drug compliance has been reported to be an important factor affecting the *H. pylori* eradication rate [21,22,23]. The adherence decreased while the severity of adverse events and number of doses increased. Compared to traditional systemic therapy with multiple-dose oral antibiotics, the ILTHPI provides a single-dose local therapy which reduced issues regarding drug absorption, tissue redistribution, liver and kidney injuries, systemic side effects, and patient compliance [11,12].

Several factors have been reported to affect the eradication rates of oral antibiotic therapies, including age, gender, BMI, serum anti-HPIgG, Type 2 DM, smoking, gastric juice pH, and peptic ulcer disease. Our study also included other factors including the DOB value for ^13^C-UBT, dyslipidemia, renal function (eGFR), anemia, and personal habits (users of alcohol, tea, coffee, non-steroid anti-inflammatory drug, steroid, and statin). The other findings of endoscopic hyperemic change and positive UFT300 of gastric cardia were also mentioned.

Tang reported that “the eradication rate of 14-day standard triple therapy was higher in ≥40-year-old patients than in <40-year-old patients (85.7% vs. 54.7%; OR: 4.58; *p* = 0.003)” [24]. Mamori et al. also reported that “the eradication rate of 7-day standard triple therapy is lower in <50-year-old-patients (OR: 0.41; 95% CI: 0.20–0.84; *p* = 0.015)” [25]. The possible reasons for the higher eradication rate are more atrophic gastric mucosa, hyposecretion of gastric acid, and better compliance of elderly patients than in younger patients. However, many studies reported no impact of age on the eradication rates of oral antibiotic therapies [21,22,23,26]. Our study also showed that the eradication rate of ILTHPI was not influenced by the age which may be due to the less impact of compliance on the ILTHPI.

Sung et al. reported that “female gender was associated with the failure of *H. pylori* eradication therapy (OR: 1.69; 95% CI: 1.12–2.55)” [26]. Other studies also reported that “female gender was associated with the failure of *H. pylori* eradication therapy due to the metronidazole and clarithromycin resistance was more common in women than men” [23,27]. However, many studies reported no impact of gender on the eradication rates of oral antibiotic therapies [21,22,24,25]. Our study also showed no influence of the gender. In certain regions with geographical differences of antibiotic resistance between women and men, the impact of gender on the eradication rate of ILTHPI should be considered.

Abdullahi et al. found that “the obese or overweight (BMI ≥ 25) non-diabetic patients showed a significantly lower *H. pylori* eradication rate than those with normal BMI (OR: 1.06; 95% CI: 1.01–1.11; *p* < 0.02) due to the larger volume of distribution of both lipophilic and hydrophilic drugs, the less active immune system and dysbiosis of stomach, and the decreased drug absorption associated with delayed gastric emptying” [28]. Other researcher reported no influence of BMI on the eradication rates of oral antibiotic therapies [29]. As shown in Table 1, the univariate analysis revealed significant influence of the BMI on the eradication rate of ILTHPI (OR: 1.15; 95% CI: 1.03–1.27; *p* = 0.011). However, the multiple logistic regression analysis showed no significant difference of the BMI (OR: 1.12; 95% CI: 0.998–1.27; *p* = 0.055). Since the ILTHPI is a local therapy leading to less impact of drug absorption, tissue redistribution, and intragastric immune reaction as compared with the traditional systemic approach [11,12]. In addition, obese subjects are at a higher risk of *H. pylori* infection [30] and *H. pylori* infection increase the risk of developing overweight or obesity [31,32]. Therefore, the overweight or obese *H. pylori* infected subjects may have more atrophic gastric mucosa and hyposecretion of gastric acid due to a longer period of *H. pylori* infection that will be favorable for the ILTHPI [12]. Furthermore, a delayed gastric emptying allows more prolonged exposure of *H. pylori* to applied antibiotics which may increase the efficacy of ILTHPI.

Shinozaki et al. reported that “high serum anti-HPIgG levels significantly predicts successful eradication of oral antibiotic therapy (OR: 2.583; 95% CI: 1.285–5.191; *p* = 0.008) due to the co-operative effect of antibiotic susceptibility and high humoral immunity” [33]. Our study showed that the eradication rate of ILTHPI was not influenced by serum anti-HPIgG. Since we excluded patient with prior usage of PPI, the intraluminal HPIgG may be degraded by pepsin when the gastric juice pH level is below 4. In addition, the humoral immunity might play a minor role for an all-in-one instant therapeutic method.

“The DOB magnitude has been correlated with the density of *H. pylori* and the grade of chronic active inflammation. The higher DOB magnitude is associated with a greater degree of successful eradication of *H. pylori* due to increased chronic active inflammation and enhanced host immune response” reported by Boltin et al. [34]. Our study revealed that the eradication rate of ILTHPI was not influenced by the DOB value for the ^13^C-UBT. Since ILTHPI provide extremely high local concentration of antibiotics which might overcome the negative impact of bacterial load and disregard the grade of gastric inflammation.

Regarding systemic diseases, several factors affecting the eradication rate of oral antibiotic therapies have been studied including the type 2 DM (T2DM), dyslipidemia, renal function (eGFR), and anemia. Only poor controlled diabetes (HbA1c ≥ 7.5%) had been reported to be a significant factor predicting the lower eradication rate of *H. pylori* (OR: 0.100; 95% CI: 0.011–0.909; *p* = 0.041) due to impaired gastric mucosal microvasculature and immunosuppressive condition of diabetes [29]. Our studies showed that the eradication rate of ILTHPI was not influenced by the T2DM, dyslipidemia, renal function (eGFR), and anemia. Due to the different administrated route and the instant efficacy of ILTHPI, there will be less impact of the intraluminal immune system. Besides, the impaired gastric mucosal microvasculature may reduce the antibiotics absorption leading to a higher intraluminal concentration of antibiotics which is beneficial for the ILTHPI.

Concerning personal habits, including the smoking, users of alcohol, tea, coffee, NSAID, steroid, and statin, only smoking had been reported to be associated with the failure of *H. pylori* oral antibiotic therapy.” The decreased gastric blood flow and secretion of mucus related to smoking could reduce the delivery of antibiotics to the gastric mucosa, thereby decreasing the efficacy of eradication therapy. In addition, smoking provokes acid secretion, resulting in decreased efficacy of acid-labile antibiotics. Furthermore, poor compliance is also an important issue in smokers” [26,35]. Our study revealed that the eradication rate of ILTHPI was not influenced by smoking and the usage of alcohol, tea, coffee, NSAID, steroid, and statin. As mentioned above, decreased gastric blood flow is favorable for the ILTHPI, while the secretion of acid and mucus can be overcome by procedures of ILTHPI [11,12].

Concerning the endoscopic findings, the crater of peptic ulcer and the inflammation of cardia might influence the eradication rate of ILTHPI due to endoscopic difficult locations for the procedures of ILTHPI including the irrigation of mucus and the application of medicaments. In addition, endoscopic hyperemic change and positive UFT300 of gastric cardia may indicate a higher bacterial load in the cardia [36], leading to failure of ILTHPI. Our study showed that the eradication rate of ILTHPI was not influenced by the presence of peptic ulcer, endoscopic hyperemic change of gastric cardia, and positive UFT300 of gastric cardia. Due to limited number in our study, we did not classify “the endoscopic findings of *H. pylori* infection regarding the presence of atrophy, intestinal metaplasia, diffuse redness, spotty redness, mucosal swelling, enlarged folds, sticky mucus, chicken skin-like nodularity, and xanthoma” [37]. However, the impact of above endoscopic findings remained to be studied.

As shown in Table 1, after multiple logistic regression analysis, the level of gastric juice pH is a crucial predicting factor for successful ILTHPI (OR: 1.30; 95% CI: 1.10–1.54; *p* = 0.002). While there are no significant influences (*p* > 0.05) of the other factors including age, gender, BMI, serum anti-HPIgG, DOB value for the ^13^C-UBT, type of disease (T2DM, dyslipidemia, value of eGFR, Anemia), personal habits (smokers, users of alcohol, tea, coffee, NSAID, steroid, statin), and endoscopic findings (gastric ulcer and/or duodenal ulcer, inflammation of cardia, positive UFT300 of cardia). Based on the results of our study, detection of gastric juice pH in ILTHPI is extremely important.

Many studies have suggested that “maintenance of intragastric pH at ideally 4 or above stabilizes the pharmacological properties of the administrated antibiotics” [38,39,40], and that “sustained control of intragastric pH at 6 or above stimulates the replication of *H. pylori* and increases the bactericidal efficacy of oral antibiotics” [38,40,41]. “Amoxicillin, clarithromycin, and metronidazole are stable at pH levels ranging from 4.0 to 7.0, 5.0 to 8.0, and 2.0 to 7.0, respectively. At gastric pH level of 2.0, the degradation half-lives of these antibiotics are 15.2 ± 0.3 h, 1.0 ± 0.04 h, and ~800 h. Clarithromycin degrades rapidly under normal gastric pH level. However, amoxicillin and metronidazole are sufficiently stable under the same conditions” [39].

The antimicrobial susceptibility of *H. pylori* was also influenced by pH level. “Ampicillin is bactericidal at pH levels of 4.5 and 7.4, but not at a pH level of 3.0, since *H. pylori* decreases the expression of its cell envelop and division genes and thus loses the ability of cell division under such level of acidity” [40]. Therefore, amoxicillin and clarithromycin are less effective at pH levels less than 4, leading to a significantly lower eradication rate of ILTHPI in patients with normal acidity (pH < 4.0) [12]. In our study, about 45.0% (27/60) in Group A, 46.0% (46/100) in Group B, and 46.3% (62/134) *H. pylori*-infected patients had levels of gastric juice pH less than 4 (Table 2). Control stomach pH at or above 4 prior to ILTHPI are strongly recommended for medicaments containing amoxicillin or clarithromycin.

Our pilot studies confirmed that *H. pylori* can be eradicated with mono-antibiotic ILTHPI. The efficacy of each mono-antibiotic LTHPI was 20% (4/20) for metronidazole, 10% (2/20) for amoxicillin, and 5% (1/20) for clarithromycin. Although no statistically significant, the eradication rate of metronidazole is higher than amoxicillin and clarithromycin. In addition, the eradication rate of ILTHPI is significantly lower in patients with mono-antibiotic ILTHPI as compared with those of triple-antibiotic ILTHPI (11.7% vs. 53.7%; *p* < 0.0001; α = 0.05, power = 1.0) (Table 3). Our results showed that the selection of optimal intraluminal medicaments is also an important factor affecting the eradication rate of ILTHPI. Besides, the efficacy of antibiotic may be quite different between the ILTHPI and traditional oral antibiotic therapies.

Amoxicillin is a bactericidal antibiotic which act by binding to a protein in transpeptidation process during bacterial cell wall synthesis and cause cell wall lysis [42,43]. Amoxicillin was bactericidal at pH range of 6.5–7.4 [39], and can reach ≥ 99.9% killing of bacteria within 12–20 h [40]. Since *H. pylori* has a doubling time of 4–6 h [44], stimulating the replication of *H. pylori* with high dose PPI or potassium-competitive acid blockers (*p*-CABs) and sustained retention of applied amoxicillin in stomach lumen for at least 4–6 h or at best 12–18 h are required to reach its best efficacy in the ILTHPI. Based on amoxicillin’s bactericidal effect and relatively lower antibiotic resistance in *H. pylori* infection [8], development of specified dual release amoxicillin without or with carriers for the ILTHPI is necessary.

Clarithromycin is a bacteriostatic antibiotic acting by binding to the 50S ribosomal subunit and inhibits RNA-dependent protein synthesis process [45]. Clarithromycin degrades rapidly under normal gastric pH level [39]. At pH 6.5, clarithromycin only caused a decrease of approximately 10% of the *H. pylori* bacterial count within 20 h [42]. Besides, there is a global trend of increasing rate of clarithromycin resistance [8], and the usage of clarithromycin mono-antibiotic ILTHPI is insufficient and unrecommended.

Metronidazole has a molecular weight of 171.15 g/mol, which is relatively a small particle size compared with other medicaments (365.4 g/mol of amoxicillin and 748 g/mol of clarithromycin) [43,45,46]. The small size allows metronidazole to act by being taken up by organisms through passive diffusion and was then reduced by electron-transport proteins to an active intermediate product. The reduced metronidazole is cytotoxic which causes DNA strand breaks, thus inhibit DNA synthesis and cell growth [46]. Not only can metronidazole display its antimicrobial effect regardless of bacterial replication, but it can also tolerate acidic environment in gastric lumen. Metronidazole is relatively stable within pH range 2.0–7.0, with a degradation half-life of more than 800 h in pH = 2.0 gastric juice sample [39]. Thus, a lower pH environment does not alter the antimicrobial effect of metronidazole. The results of our study indicated that metronidazole is suitable for the ILTHPI (Table 3). Before the development of a novel acid-stable antimicrobial agent for ILTHPI, we recommend metronidazole-containing medicaments for the ILTHPI.

To the best of our knowledge, there has so far been no specified medicament for the ILTHPI. “To enhance the efficacy of ILTHPI, we suggested the development of more specified intraluminal antibiotics or antimicrobial agents without or with carriers (such as solution or suspension formula, dual-release medicament, antibiotic-loaded polymers, antibiotic-loaded biodegradable microspheres, or antibiotic-loaded nanoparticles), use of bismuth, combined antibiotics with synergistic effect, use of susceptibility-guided ILTHPI via molecular stool testing, and the supplementation of suitable probiotics” [11,12].

Asides from the efficacy of antibiotics, “at pH level of less than 4, the conformational change of mucin facilitates cross-links among mucin macromolecules through hydrophobic interactions [47], which in turn lead to a solution-to-gel transition (sol-gel) [48]. When exposing gastric mucus to pH levels below 4, the gel-forming mucus layer serves as a critical barrier for blocking the diffusion of antibiotic particles applied from the gastric lumen, and plays a role in ensuring the colonization of *H. pylori* in the adherent mucus gel and its underlying epithelial layer [49], which is unfavorable for both the irrigation of gastric mucus and the application of antimicrobial agents” [12]. Whether the use of other mucolytic agents (such as ambroxol, carbocysteine, or erdosteine agents) could be more suitable for patients with gastric juice pH below 4 remains to be investigated. “The inventing of suitable devices specifically tailored to the ILTHPI will also be beneficial to shorten the duration of the ILTHPI and improve its eradication rate. The specified devices may include a more effective pumping machine, a more powerful shower nozzle for irrigation, and a specifically designed sprayer nozzle for the application of medicaments” [11,12].

Current guidelines suggest avoiding the reuse of clarithromycin due to the secondary resistance of clarithromycin after failure of first line multiple-dose oral antibiotic therapy [2,6,7]. Whether single-dose antibiotic applied in ILTHPI has a potential to increase the resistance rate of antibiotic and decrease the eradication rate of subsequent oral antibiotic therapy remains unknown. Table 4 showed that the usage of 14-day clarithromycin triple therapy for patients failed clarithromycin containing mono-antibiotic ILTHPI can still achieve an optimal eradication rate (94.7% [18/19]; 95% CI: 73.5% to 99.9%). Meanwhile, the ITT and PP eradication rates of 14-day clarithromycin triple therapy are higher for patients with failed ILTHPI than those without ILTHPI (ITT: 94.7% vs. 80.4%; PP: 94.7% vs. 84.1%) (Table 5). The ITT and PP eradication rates are also higher for patients with failed ILTHPI than those without ILTHPI in subgroups of 14-day high dose dual therapy and 14-day metronidazole-based triple therapy (Table 5). Our study revealed that reuse of the same antibiotics applied in the ILTHPI do not decrease the eradication rate of subsequent oral antibiotic therapies, which indicated that a single-dose antibiotic applied in the ILTHPI may not induce a secondary antibiotic resistance as compared to the multiple-dose oral antibiotic therapies. As a local therapy, the ILTHPI may have a potential to diminish the increasing rate of global antibiotic resistance. However, due to the limited number of patients in our study, further studies with large number of patients and more investigations of other antibiotics, including the antibiotic susceptibility tests, are recommended.

## 5. Conclusions

In conclusion, the level of gastric juice pH is a crucial factor affecting the eradication rate of ILTHPI. The detection of gastric juice pH and selection of optimal intraluminal medicaments for the ILTHPI are important. The eradication rate of single antibiotics (amoxicillin, clarithromycin, or metronidazole) for the ILTHPI is low. To improve the eradication rate of ILTHPI, further studies with combined antibiotics, perhaps metronidazole-containing medicaments, is recommended.

## Figures and Tables

**Table 1:** Logistic regression analysis to factors affecting the ILTHPI.

Factors	Univariate Analysis			Multivariate Analysis		
	OR	95% CI	*p*-Value	OR	95% CI	*p*-Value *
Age (years)	1.02	0.98, 1.07	0.282	1.03	0.97, 1.08	0.322
Male (ref. Female)	1.97	0.86, 4.49	0.107			
Body mass index	1.15	1.03, 1.27	0.011	1.12	0.998, 1.27	0.055
Serum HPIgG	0.93	0.75, 1.16	0.534			
^13^C-UBT (DOB)	1.00	0.98, 1.03	0.689	1.00	0.98, 1.03	0.739
Type 2 DM (ref. non-T2DM)	1.24	0.45, 3.42	0.681	0.77	0.23, 2.63	0.680
Dyslipidemia (ref. non-DL)	1.29	0.50, 3.28	0.598			
eGFR	0.99	0.97, 1.01	0.274			
Anemia (ref. non-Anemia)	0.85	0.20, 3.62	0.827			
Smokers (ref. non-smokers)	0.81	0.48, 1.39	0.450	1.14	0.61, 2.16	0.678
Alcohol users	0.76	0.39, 1.48	0.416			
Tea users	0.88	0.68, 1.14	0.328			
Coffee users	0.88	0.70, 1.12	0.307			
NSAID users	0.73	0.28, 1.93	0.528			
Steroid users	2.69	0.27, 26.8	0.400			
Statin users	0.70	0.22, 2.28	0.559			
Gastric juice pH	1.35	1.16, 1.58	0.0001	1.30	1.10, 1.54	0.002
PUD (ref. non-PUD)	0.59	0.24, 1.46	0.257	0.70	0.25, 1.96	0.495
Carditis (ref. non-Carditis)	1.54	0.68, 3.49	0.304			
Positive UFT300 of cardia	1.95	0.66, 5.72	0.225			

ILTHPI, intraluminal therapy for *Helicobacter pylori* infection; OR, odds ratio; CI, confidential interval; HPIgG, Anti-*Helicobacter pylori* IgG; ^13^C-UBT, ^13^C-urea breath test; DOB, delta over baseline; DM, diabetes mellitus; DL, dyslipidemia; eGFR, estimated glomerular filtration rate; NSAID, non-steroid anti-inflammatory drug; PUD, peptic ulcer disease; UFT, ultrafast 300 quick test. * A Bonferroni-corrected threshold of 0.05/7 = 0.007.

**Table 2:** Clinical characteristics of *Helicobacter pylori* infected patients.

Characteristics	Group A ^†^	Group B ^†^	Group C ^‡^
	(*n* = 60)	(*n* = 100)	(*n* = 134)
Age (years, mean ± SD/range) *	51.6 ± 11.7 (26–73)	52.1 ± 10.3 (24–74)	51.8 ± 11.5 (20–75)
Gender (M/F) *	27/33	47/53	62/72
NSAID ingestion *	12 (20.0%)	21 (21.0%)	26 (19.4%)
Smoking *	10 (16.7%)	17 (17.0%)	24 (17.9%)
Alcohol consumption *	5 (8.3%)	7 (7.0%)	11 (8.2%)
Ingestion of tea *	17 (28.3%)	30 (30.0%)	43 (32.1%)
Ingestion of coffee *	25 (41.7%)	39 (39.0%)	52 (38.8%)
BMI (kg/m^2^, mean ± SD/range) *	25.3 ± 4.6	25.9 ± 4.4	25.6 ± 4.5
	(17.7–39.8)	(17.5–36.5)	(17.4–38.5)
Endoscopic Findings *			
Normal	10 (16.7%)	15 (15.0%)	18 (13.4%)
Gastritis	50 (83.3%)	85 (85.0%)	116 (86.6%)
(antrum/corpus/cardia)	(23/41/32)	(39/66/57)	(54/84/74)
Peptic ulcer disease	15 (25.0%)	28 (28.0%)	37 (27.6%)
Gastric Juice pH *			
pH ≤ 3.5	27 (45.0%)	46 (46.0%)	62 (46.3%)
pH 4–5.5	4 (6.7%)	9 (9.0%)	13 (9.7%)
pH ≥ 6.0	29 (48.3%)	45 (45.0%)	59 (44.0%)

**^†^** Group A, intraluminal mono-antibiotic therapy; **^†^** Group B, intraluminal triple antibiotic therapy; **^‡^** Group C, oral antibiotic therapy without ILTHPI; SD, standard deviation; NSAID, non-steroid anti-inflammatory drug. BMI, body mass index. * *p* > 0.05 for all clinical characteristics among Group A, Group B, and Group C.

**Table 3:** Medicaments affecting the efficacy and adverse event of ILTHPI.

Medicaments	Patients Number (Lost to Follow Up)	Eradication Rate	Adverse Event
Mono-antibiotic	60 (0)	7/60 (11.7%) *	1/60 (1.7%) **
Amoxicillin	20	2/20 (10%)	0/20 (0%)
Clarithromycin	20	1/20 (5%)	0/20 (0%)
Metronidazole	20	4/20 (20%)	1/20 (5%)
Triple antibiotic	100 (5)	51/95 (53.7%) *	6/100 (6%) **

ILTHPI, intraluminal therapy for *Helicobacter pylori* infection. * *p* < 0.0001 for the eradication rate comparing medicament containing mono-antibiotic (amoxicillin, clarithromycin or metronidazole) versus triple antibiotic (amoxicillin, clarithromycin, and metronidazole). ** *p* = 0.257 for the adverse event comparing mono-antibiotic therapy versus triple antibiotic therapy.

**Table 4:** The overall eradication rate of intraluminal therapy plus oral antibiotic therapy.

ILTHPI (Success No.)	Oral Antibiotic Therapy (No.)	Eradication Rate ^†^ (ITT = PP)	Overall Eradication Rate (ILTHPI Plus Oral Antibiotic Therapy)
Amoxicillin (2)	HDDT-14 (18)	16/18 (88.9%)	18/20 (90.0%)
Clarithromycin (1)	CTT-14 (19)	18/19 (94.7%)	19/20 (95.0%)
Metronidazole (4)	MTT-14 (16)	12/16 (75.0%)	16/20 (80.0%)

ILTHPI: Intraluminal therapy for *Helicobacter pylori* infection. ITT, intention-to-treat; PP, per-protocol. ^†^ Eradication Rate for patients failed ILTHPI: HDDT-14, high dose dual therapy for 14 days; CTT-14, clarithromycin triple therapy for 14 days; MTT-14, metronidazole-based triple therapy for 14 days.

**Table 5:** The efficacy of oral antibiotic therapy for patients failed ILTHPI and without ILTHPI.

ILTHPI	Oral Antibiotic Therapy	Eradication Rate ^†^ (Failed ILTHPI)	ITT Eradication Rate (without ILTHPI)	PP Eradication Rate (without ILTHPI)
Amoxicillin	HDDT-14 *	16/18 (88.9%)	38/45 (84.4%)	38/44 (86.4%)
Clarithromycin	CTT-14 *	18/19 (94.7%)	37/46 (80.4%)	37/44 (84.1%)
Metronidazole	MTT-14 *	12/16 (75.0%)	31/43 (72.1%)	31/42 (73.8%)

ILTHPI, intraluminal therapy for *Helicobacter pylori* infection; ITT, intention-to-treat; PP, per-protocol; HDDT-14, high dose dual therapy for 14 days; CTT-14, clarithromycin triple therapy for 14 days; MTT-14, metronidazole-based triple therapy for 14 days. ^†^ Eradication Rate: ITT = PP. * *p* > 0.05 for the ITT and PP eradication rates between patients failed ILTHPI and without ILTHPI.

## Data Availability

Not applicable.
